# Narrow-band imaging (NBI) for improving the assessment of vocal fold leukoplakia and overcoming the umbrella effect

**DOI:** 10.1371/journal.pone.0180590

**Published:** 2017-06-29

**Authors:** H. Klimza, J. Jackowska, M. Tokarski, K. Piersiala, M. Wierzbicka

**Affiliations:** 1 Department of Otolaryngology, Head and Neck Surgery, Poznań University of Medical Sciences, Poznań, Poland; 2 Student Research Group at the Department of Otolaryngology, Head and Neck Surgery Poznań University of Medical Sciences, Poznań, Poland; Texas A&M University, UNITED STATES

## Abstract

**Background:**

It is crucial to find a balance between functional and oncological outcome when choosing an adequate method for the management of vocal fold leukoplakia. Therefore, a detailed examination is a milestone in the decision-making process.

**Aim:**

To examine whether narrow-band imaging (NBI) can be helpful in vocal fold assessment in the case of leukoplakia and how to overcome the “umbrella effect”- understood as the submucosal vascular pattern hidden under the plaque.

**Material and methods:**

Prospective cohort of 41 consecutive patients. Inclusion criteria: vocal fold leukoplakia, no previous procedures (surgery, radiotherapy), and preoperative endoscopy with an optical filter for NBI. Two groups: “suspicious” and “normal”, according to the submucosal microvascular pattern of peripheral regions of the mucosa surrounding the plaque, were distinguished. Patients were qualified for a full-thickness or partial-thickness biopsy, respectively. Criteria defining suspected characters were well-demarcated brownish areas with scattered brown spots corresponding to type IV, Va, Vb, and Vc NI classifications.

**Results:**

In 22/41 (53.7%) patients with “suspected” microvascular pattern, full-thickness biopsy was performed. Moderate and severe dysplasia was revealed in 15 type IV and 7 type Va NI patients. In 19/41 (46.3%) patients with proper NBI vessel pattern treated by partial-thickness biopsy, hyperkeratosis was diagnosed. There was a strong correlation between the NBI pattern and final histology: Chi2 (2) = 41.0 (p = 0.0000).

**Conclusion:**

The results demonstrate that NBI endoscopic assessment of the submucosal microvascular pattern of mucosa surrounding the plaque can be an effective method to categorise the risk in vocal fold leukoplakia prior to treatment.

## Introduction

Laryngeal leukoplakia is defined as a clinical finding of whitish patches or plaques on the epithelium, which is associated with tobacco smoking, alcohol abuse, voice misuse, viral infection, and laryngopharyngeal reflux [1]. There are multiple histological diagnoses of vocal fold leukoplakia including: benign, premalignant, and malignant lesions [2,3]. According to the WHO, the division of laryngeal leukokeratosis is as follows: 1. Hyperplasia; 2. Mild dysplasia; 3. Moderate dysplasia; 4. Severe dysplasia; and 5. Carcinoma *in situ* [4].

Predominantly, patients with vocal fold leukoplakia suffer from hoarseness and expect restoration of their voice after an appropriate therapy. In some cases, patients do not experience any voice disturbances. For this reason, the preservation of vocal fold function is the main goal of an adequate and modern leukoplakia treatment [3]. However, we have to simultaneously highlight the oncological principle and answer the crucial question: what is hidden under the leukoplakia?

Currently, there is no ideal treatment schedule for vocal fold leukoplakia. The main methods include: conservative therapy (strict voice rest, inhaled glucocorticoid therapy and empiric PPI), stripping of mucosa, surgical biopsies by cold instrument or laser CO_2_, partial subepithelial or subligamental cordectomy or even radiation [1,5] The choice of method is crucial to find a balance between a functional and oncological outcome.

As a result, a thorough examination is a milestone in the decision-making process. In our study, we want to discuss the value of Narrow Band Imaging (NBI) in the preoperative assessment of patients with vocal fold leukoplakia. NBI allows the identification of superficial capillaries and neoangiogenesis in abnormal mucosa [6]. NBI highlights pathological vessels in precancerous and cancerous lesions by enhancing the contrast of the mucosal epithelium and submucosal vessel loops. However, there is a well-known phenomenon, the “umbrella effect”, which reflects the submucosal vascular pattern hidden under the hyperkeratotic plaque. Thereby, we want to examine how to improve the assessment of vocal fold leukoplakia. To overcome the umbrella effect, the NBI was used to categorise submucosal microvascular pattern surrounding the plaque and served to determine whether NBI can be an adequate method to guide the voice-sparing depth of incision. The aim of this study was to assess the accuracy of preoperative evaluation of vocal fold leukoplakia by means of NBI in comparison with final histology and follow-up outcomes. The NBI was used to categorise the submucosal microvascular pattern and served to add a new information compared to the description of suspicious vocal fold lesions by Ni et al. [7].

## Material and methods

Forty one consecutive patients with vocal fold leukoplakia, 28 (68.3%) males and 13 (31.7%) females, aged 35–80 years (mean 61.1 years), were included between April 2012 and December 2015 in the prospective study conducted in the Department of Otolaryngology, Head and Neck Surgery of Poznan University of Medical Sciences, which is a tertiary referral centre. In these patients, 6/41 (14.6%) had bilateral leukoplakia and 35/41 (85.4%) unilateral leukoplakia. The histological structure of laryngeal leukokeratosis was assessed according to the WHO classification [8]. Age, sex, medical history, preoperative laryngoscopy in WL and NBI, and routine histology were analysed. All patients fulfilled the inclusion criteria: the presence of vocal fold leukoplakia, no previous procedures (surgery, radiotherapy), and preoperative endoscopy with an optical filter for NBI. Two groups were distinguished: “suspicious” and “normal”, divided according to submucosal microvascular pattern of mucosa surrounding the plaque. Patients were qualified for a full-thickness or partial-thickness biopsy, respectively. Ethical approval: All procedures performed in studies involving human participants were in accordance with the ethical standards of the institutional and national research committee and with the 1964 Helsinki declaration and its later amendments or comparable ethical standards. The study protocol was approved by Bioethics Committee of Poznan University of Medical Sciences.

Informed consent: Informed written consent was obtained from all individual participants included in the study.

### The preoperative work-up and treatment

Every patient underwent trans-nasal flexible video-endoscopy (video processor with integrated LED light source, model CV-170 with HD, ENF-VH, Olympus Corp, Tokyo, Japan) with the optical filter for NBI [9] and video-stroboscopic laryngeal examination before decision-making. The rhinolaryngo-videoendoscopic and videostrobscopic examinations were performed in an outpatient setting. The larynx was examined by white light view using trans-nasal flexible video-endoscope and 2 doctors (M.W and J.J). The vocal fold leukoplakia was assessed paying special attention to texture, colour, size, redness, symmetry and thickness. Afterwards, by pushing a fingertip control switch on the endoscope, the NBI view was obtained. The submucosal microvascular patterns that surrounded the vocal fold leukoplakia were assessed. By using NBI, the vessels of the proximal part of the vocal fold, distal part of the vocal fold, subglottic region and laryngeal ventricle were exposed. The diagnostic criteria defining suspected characters of lesions in NBI endoscopy were as follows: well-demarcated brownish areas with scattered brown spots which correspond to type IV, Va, Vb, and Vc classification according to Ni et al. [7]. If such lesions surrounded the vocal fold leukoplakia S1 and S2 Figs, the patient was called as “suspicious” and qualified for more aggressive: full-thickness biopsies. The full-thickness biopsy included four layers of the vocal fold (epithelium, lamina propria, ligamentum vocalis and vocalis muscle) only at the site of leukoplakia. Patients with plaques not surrounded by suspicious mucosa were called “normal” and qualified for partial-thickness biopsies. The partial-thickness biopsy included only three layers of the vocal fold (epithelium, lamina propria and ligamentum vocalis) only at the site leukoplakia. All procedures were performed under general anaesthesia, based on information obtained in NBI and videostroboscopy. Eventually, the surgical specimens were sent for routine histopathological examination.

The main predictive variables taken into consideration were mucosal and submucosal vessel patterns according to NI classification and final histology.

The additional variables were patient’s age, sex, duration of hoarseness, smoking, and details from videostroboscopy.

The primary outcome measure was the correlation between the NI classification of the mucosa surrounding leukoplakia and the results of the histological examination.

## Results

Out of the 41 patients with leukoplakia qualified for surgery, 22 (53.7%) had a “suspicious” microvascular NBI pattern in the mucosa surrounding the leukoplakia plaque. Microvascular network corresponded to type IV and type Va NI classification in 15/22 and in 7/22 patients, respectively. In this group, a full-thickness biopsy was performed. The exact location of the divergent microvascular pattern was as follows: the proximal part of the vocal fold 9/22 (40.9%), the distal part of the vocal fold 6/22 (27.3%), the subglottic region 6/22 (27.3%), and the laryngeal ventricle 9/22 (40.9%). A “Normal” NBI pattern in epithelium adjacent to leukoplakia plaques was seen in 19/41 (46.3%) patients; thus, only partial-thickness biopsies were performed.

In all patients, the microvascular NBI pattern was compared with the final histology. Out of 22 “suspected” patients with full-thickness biopsy, in 15 with type IV, moderate dysplasia, and in 7 with type Va, severe dysplasia, were observed. In 19/41 patients with normal NBI vessel patterns treated by partial-thickness biopsy, hyperkeratosis was diagnosed. There was a significant correlation between the NBI pattern of mucosa surrounding the leukoplakia plaque and the final histological result: Chi2 (2) = 41.0 (p = 0.0000). The localisation of leukoplakia has no impact on histology Chi2 (2) = 1.83 (p = 0.3997).

The mean age of patients with mild/moderate, severe dysplasia and hyperkeratosis was 63.8, 59.3 and 60.7 years, respectively. There was no interrelation between the patient’s age and histology (Kruskal Wallis test, p = 0.211).

Hyperkeratosis, mild/moderate and severe dysplasia occurred in 35.7%, 25.0% and 39.3% of males, respectively. Hyperkeratosis was observed in 69.2% and mild dysplasia in 30.8% of women. There was a significant correlation between the histology and sex (p = 0.025), as severe dysplasia occurred only in men (n = 11).

The mean duration of hoarseness in patients with mild/moderate, severe dysplasia and hyperkeratosis was 5.3, 5.6, and 2.7 months, respectively. There was a correlation between histology and the length of the patient’s hoarseness (Kruskal Wallis test p = 0.000). The patients with moderate and severe dysplasia and “suspicious” vessel patterns had a longer duration of hoarseness compared to patients with hyperkeratosis.

The majority of patients 27/41 (65.6%) were heavy smokers. There was a significant correlation between the smoking habit and histology (Chi2 (2) = 24,61; p = 0.000). In all non-smoking patients there was only hyperkeratosis.

In all patients, a vidoelaryngostroboscopy examination was performed. In each case, a limit of mucosal vibration in the region of a suspicious lesion was observed.

The results of the study are summarized in S1 Table.

## Discussion

For decades, laryngeal leukoplakia has been regarded as a very controversial issue, concerning the classification, histological assessment and treatment of these lesions [5,10]. Currently, there is no ideal histological grading system for premalignant laryngeal lesions. Nowadays, the Ljubljana classification (four-grade system), WHO dysplasia system (five grade system) and SIN-system (squamous intraepithelial neoplasia) are used for the assessment of laryngeal leukoplakia [11]. In 2014, Gale et al. proposed a new classification of laryngeal precursor lesions as follows: low-grade SIL (squamous intraepithelial lesions), high-grade SIL and CIS (carcinoma *in situ*) [12, 13]. These authors believe that the three-grade classification will be widely used by pathologists in the future. In our paper, we used the WHO dysplasia system for the assessment of laryngeal leukokeratosis.

The treatment of vocal fold leukoplakia, potentially carrying dysplasia, is still the subject of debate. After thorough examination of the white plaque, long-term observation should be conducted in selected cases [14]. According to Tuan-Jen Fang et al., in suspected benign lesions, observation or superficial excision is preferred; however, deeper excision is reserved for cases with malignant potential [5]. The vocal leukoplakia with the appearance of an irregular surface and redness should be managed more aggressively than those without these features, especially in older patients [5].

The most important factor is a thorough identification of adverse prognostic factors in vocal fold leukoplakia before treatment. It helps to avoid unnecessary surgery and achieve better treatment efficacy [1]. We aimed to find a method, which will reliably guide the voice-sparing depth of incision. Therefore, we wanted to examine how to improve the assessment of vocal fold leukoplakia by using the NBI to categorise the submucosal microvascular pattern surrounding the plaque. Thus, in this study, we have made an effort to add new information to the method described by Ni et al. [7], we overcame the umbrella effect, and well-known disturbances of translucency in hyperkeratotic leukoplakia plaques, shadowing the microvascular pattern. NBI was recommend as a useful tool in distinguishing between low-grade and high-grade dysplasia by Watanabe et al. [15]. According to Xinmeng et al., the NBI image of vocal fold leukoplakia has its own character, presented as a white colour and hyperplastic epithelial lesions appeared as a slightly brownish colour [16]. However, the limitation of this method is hyperkeratotic white plaques covering proper lesions; thus, we focused on the assessment of microvascular patterns in the surrounding mucosa.

In the current literature, we found few papers devoted to NBI imaging and hyperkeratotic mucosa. According to Stanikowa et al. [17] the correlation between inappropriate vessel pattern around laryngeal leukoplakia and histological examination are consistent in 88.89% of cases. In our study, the vessel pattern in NBI had a good correlation with final histology and confirmed the findings of others [17]. We described and recommended the use of NBI endoscopy for the assessment of edges of the white plaque and surrounding mucosa to choose the appropriate treatment.

Regarding the depth of incision, Annelienke et al. [4] believe that moderate dysplasia is connected with a much higher risk of transformation to malignancy than was previously believed. Therefore, it requires more careful observation or even more aggressive treatment. On the other hand, Kono et al. recommended phonomicrosurgical resection via the submucosal infusion technique as a good method of treatment in patients with vocal fold leukoplakia [18]. According to our experience, the NBI vessel pattern and categorisation of NBI image to “normal” and “suspicious” have the potential to guide the depth of incision in vocal fold leukoplakia.

The limitations of this study were as follows: a limited number of participants, a very subjective evaluation of the vocal folds by means of NBI and a relatively long learning curve for this technique. The categorisation of “normal” and “suspicious" can be useful in qualifying the plaque for a full-thickness or partial-thickness biopsy, which allows the histological evaluation to return with a verified pre-operative diagnosis. Conversely, the categorisation of leukoplakia based on NBI for further observation should be carefully monitored with close follow-up.

## Conclusion

The use of NBI endoscopy improves the diagnostics of laryngeal leukoplakia due to assessment of intraepithelial capillary loops in the mucosa surrounding the plaque. If this mucosa is not suspected in NBI examination, with normal proximal and distal vocal fold vessel pattern around the leukoplakia, there is a low risk of high-grade dysplasia and cancer *in situ* underneath the plaque. The NBI endoscopy is useful in decision making concerning the depth of leukoplakia plaque sampling.

## Supporting information

S1 Fig Leukoplakia in white light (WL). (TIF)

S2 Fig Leukoplakia in NBI–pathological distal vessels at the site of leukoplakia. (TIF)

S1 Table Summary of the results. (DOCX)
