# Supplementary figures and images for: Narrow-band imaging (NBI) for improving the assessment of vocal fold leukoplakia and overcoming the umbrella effect
Source: PLoS One. 2017 Jun 29;12(6):e0180590. doi: 10.1371/journal.pone.0180590 (PMC5491250; doi:10.1371/journal.pone.0180590)

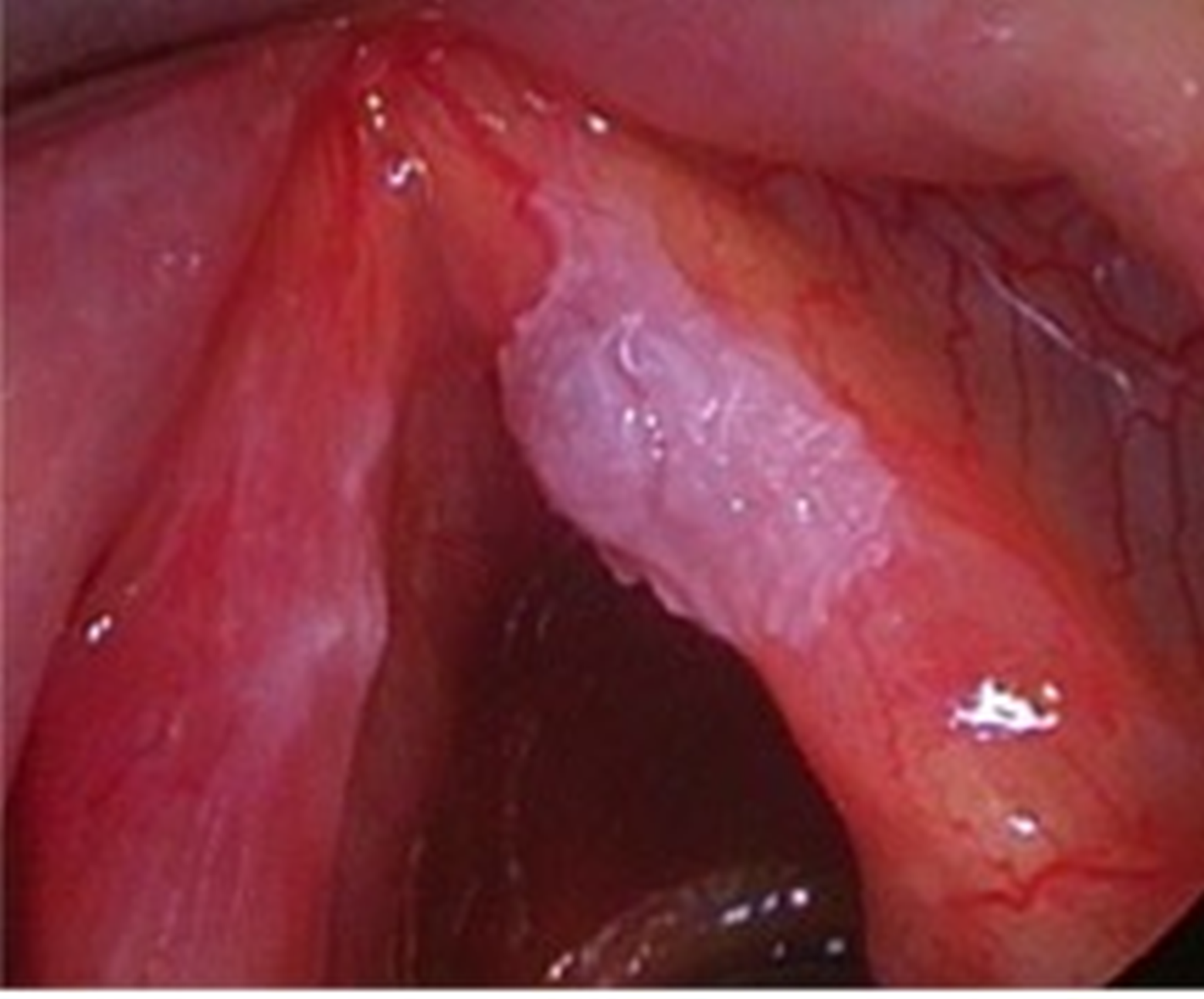

Supplement: S1 Fig — (TIF) [file pone.0180590.s001.tif]

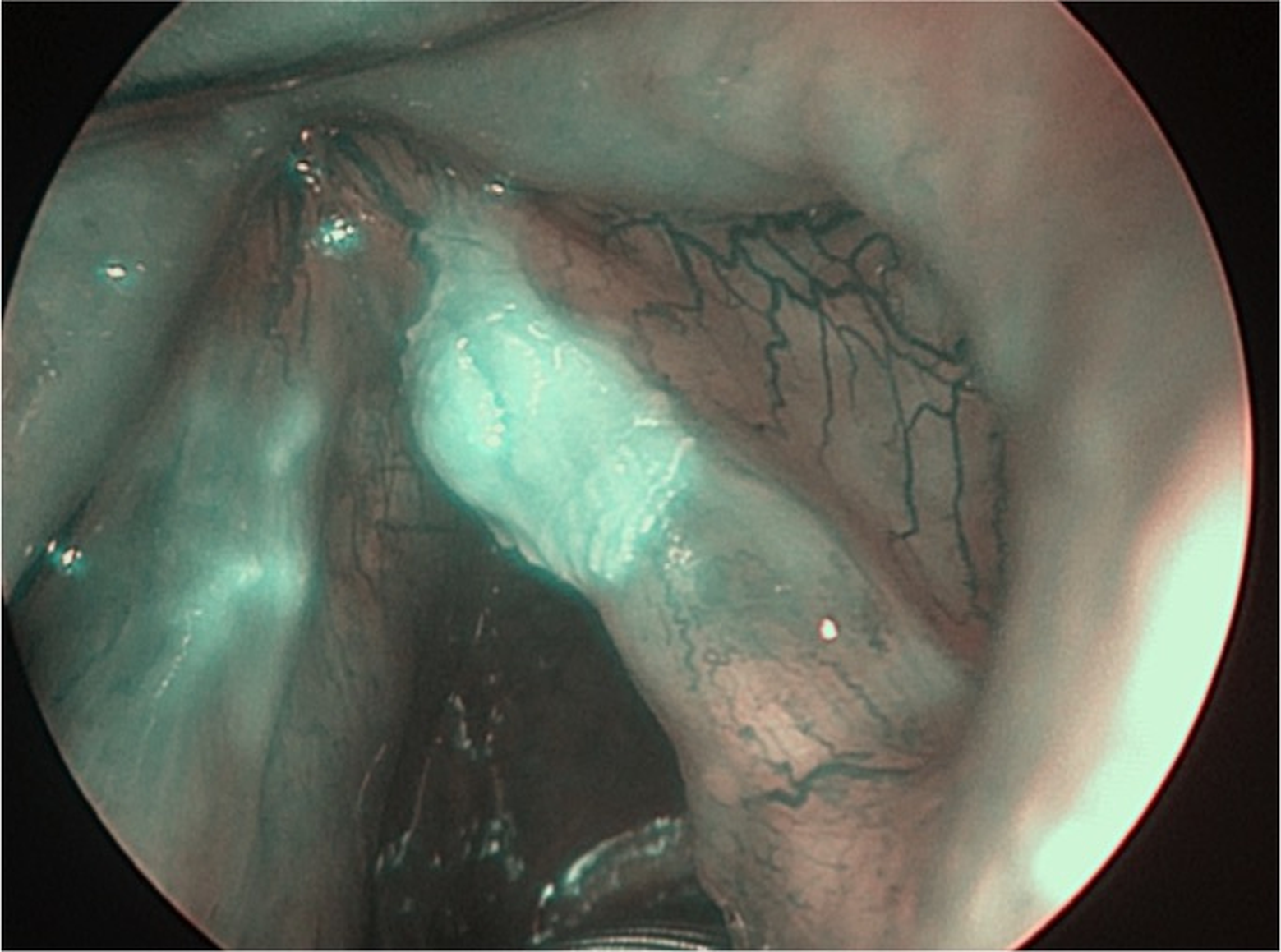

Supplement: S2 Fig — (TIF) [file pone.0180590.s002.tif]
