# Supplementary material for: Narrow-band imaging (NBI) for improving the assessment of vocal fold leukoplakia and overcoming the umbrella effect
Source: PLoS One. 2017 Jun 29;12(6):e0180590. doi: 10.1371/journal.pone.0180590 (PMC5491250; doi:10.1371/journal.pone.0180590)
Supplement: S1 Table — (DOCX) [file pone.0180590.s003.docx]

|  | **Age** | **Sex** | **Unilateral/ bilateral** | **The assessment of vessels by NBI in the area of leukoplakia** | | | | **Biopsy^a,b^** | **Histology** | **Follow up** | **Duration of hoarseness (months)** | **Heavy smoker** |
| --- | --- | --- | --- | --- | --- | --- | --- | --- | --- | --- | --- | --- |
|  |  |  |  | **Proximal** | **Distal** | **Subglottic** | **Pocket laryngeal** |  |  |  |  |  |
| 1 | 61 | m | unilateral | no | no | No | no | partial | hyperkeratosis | no | 3 | no |
| 2 | 59 | m | unilateral | no | no | No | no | partial | hyperkeratosis | no | 2 | no |
| 3 | 57 | m | unilateral | IVa | IVa | No | no | full | severe dysplasia | no | 5 | yes |
| 4 | 70 | f | bilateral | IVa | no | No | no | full | moderate dysplasia | no | 3 | yes |
| 5 | 70 | f | bilateral | no | no | No | no | partial | hyperkeratosis | no | 4 | yes |
| 6 | 57 | m | unilateral | Va | Va | Va | Va | full | severe dysplasia | yes | 2 | yes |
| 7 | 64 | m | unilateral | no | no | No | no | partial | hyperkeratosis | no | 1 | no |
| 8 | 67 | f | unilateral | no | no | No | no | partial | hyperkeratosis | no | 1 | no |
| 9 | 47 | m | unilateral | no | no | No | IVa | full | moderate dysplasia | no | 2 | yes |
| 10 | 60 | m | unilateral | no | no | Va | Va | full | severe dysplasia | no | 10 | yes |
| 11 | 61 | m | unilateral | no | no | No | no | partial | hyperkeratosis | no | 3 | yes |
| 12 | 59 | m | unilateral | no | no | No | no | partial | hyperkeratosis | no | 4 | no |
| 13 | 57 | m | unilateral | IVa | IVa | No | no | full | severe dysplasia | no | 4 | yes |
| 14 | 70 | f | bilateral | IVa | no | No | no | full | moderate dysplasia | no | 7 | yes |
| 15 | 70 | f | bilateral | no | no | No | no | partial | hyperkeratosis | no | 1 | no |
| 16 | 64 | m | unilateral | Va | Va | Va | Va | full | severe dysplasia | yes | 7 | yes |
| 17 | 57 | m | unilateral | no | no | No | no | partial | hyperkeratosis | no | 5 | yes |
| 18 | 67 | f | unilateral | no | no | No | no | partial | hyperkeratosis | no | 4 | no |
| 19 | 47 | m | unilateral | no | no | No | IVa | full | moderate dysplasia | no | 3 | yes |
| 20 | 64 | m | unilateral | no | no | Va | Va | full | severe dysplasia | no | 9 | yes |
| 21 | 61 | m | unilateral | no | no | No | no | partial | hyperkeratosis | no | 2 | no |
| 22 | 59 | m | unilateral | no | no | No | no | partial | hyperkeratosis | no | 5 | no |
| 23 | 57 | m | unilateral | IVa | IVa | No | no | full | severe dysplasia | no | 3 | yes |
| 24 | 70 | f | bilateral | IVa | no | No | no | full | moderate dysplasia | no | 8 | yes |
| 25 | 70 | f | bilateral | no | no | No | no | partial | hyperkeratosis | no | 1 | no |
| 26 | 60 | m | unilateral | Va | Va | Va | Va | full | severe dysplasia | no | 7 | yes |
| 27 | 57 | m | unilateral | no | no | No | no | partial | hyperkeratosis | no | 4 | no |
| 28 | 67 | f | unilateral | no | no | No | no | partial | hyperkeratosis | no | 1 | yes |
| 29 | 47 | m | unilateral | no | no | No | IVa | full | moderate dysplasia | no | 5 | yes |
| 30 | 60 | m | unilateral | no | no | Va | Va | full | severe dysplasia | no | 2 | yes |
| 31 | 70 | m | unilateral | IVa | no | No | no | full | moderate dysplasia | no | 7 | yes |
| 32 | 75 | m | unilateral | no | no | No | IVa | full | moderate dysplasia | no | 6 | yes |
| 33 | 65 | m | unilateral | Va | Va | No | no | full | severe dysplasia | no | 8 | yes |
| 34 | 61 | m | unilateral | no | no | No | no | partial | hyperkeratosis | no | 2 | no |
| 35 | 69 | f | unilateral | no | no | No | no | partial | hyperkeratosis | no | 5 | no |
| 36 | 61 | m | unilateral | no | IVa | No | no | full | moderate dysplasia | no | 2 | yes |
| 37 | 57 | f | unilateral | IVa | no | No | no | full | moderate dysplasia | no | 7 | yes |
| 38 | 56 | f | unilateral | no | no | No | no | partial | hyperkeratosis | no | 1 | no |
| 39 | 44 | m | unilateral | IVa | IVa | No | no | full | severe dysplasia | no | 5 | yes |
| 40 | 35 | f | unilateral | no | no | No | no | partial | hyperkeratosis | no | 2 | yes |
| 41 | 80 | m | unilateral | no | IVa | No | no | full | moderate dysplasia | no | 8 | yes |
